# DNA tetrahedron-mediated immune-sandwich assay for rapid and sensitive detection of PSA through a microfluidic electrochemical detection system

**DOI:** 10.1038/s41378-021-00258-x

**Published:** 2021-04-25

**Authors:** Dezhi Feng, Jing Su, Yi Xu, Guifang He, Chenguang Wang, Xiao Wang, Tingrui Pan, Xianting Ding, Xianqiang Mi

**Affiliations:** 1 Key Laboratory of Functional Materials for Informatics, Shanghai Institute of Microsystem and Information Technology, Chinese Academy of Sciences, 200050 Shanghai, China; 2 Shanghai Advanced Research Institute, Chinese Academy of Sciences, 201210 Shanghai, China; 3 University of Chinese Academy of Sciences, 100049 Beijing, China; 4 State Key Laboratory of Oncogenes and Related Genes, Institute for Personalized Medicine, School of Biomedical Engineering, Shanghai Jiao Tong University, 200030 Shanghai, China; 5 School of Life Sciences, Shanghai University, 200444 Shanghai, China; 6 Shenzhen Institutes of Advanced Technology, Chinese Academy of Science, 518055 Shenzhen, China; 7 CAS Center for Excellence in Superconducting Electronics, (CENSE), 200050 Shanghai, China; 8 Key Laboratory of Systems Health Science of Zhejiang Province, Hangzhou Institute for Advanced Study, University of Chinese Academy of Sciences, Chinese Academy of Sciences, 310024 Hangzhou, China

**Keywords:** Biosensors, Microfluidics, Nanostructures

## Abstract

Prostate-specific antigen (PSA) is the most widely used biomarker for the early diagnosis of prostate cancer. Existing methods for PSA detection are burdened with some limitations and require improvement. Herein, we developed a novel microfluidic–electrochemical (μFEC) detection system for PSA detection. First, we constructed an electrochemical biosensor based on screen-printed electrodes (SPEs) with modification of gold nanoflowers (Au NFs) and DNA tetrahedron structural probes (TSPs), which showed great detection performance. Second, we fabricated microfluidic chips by DNA TSP-Au NF-modified SPEs and a PDMS layer with designed dense meandering microchannels. Finally, the μFEC detection system was achieved based on microfluidic chips integrated with the liquid automatic conveying unit and electrochemical detection platform. The μFEC system we developed acquired great detection performance for PSA detection in PBS solution. For PSA assays in spiked serum samples of the μFEC system, we obtained a linear dynamic range of 1–100 ng/mL with a limit of detection of 0.2 ng/mL and a total reaction time <25 min. Real serum samples of prostate cancer patients presented a strong correlation between the “gold-standard” chemiluminescence assays and the μFEC system. In terms of operation procedure, cost, and reaction time, our method was superior to the current methods for PSA detection and shows great potential for practical clinical application in the future.

## Introduction

Prostate cancer is one of the most common malignant tumors in the male population in the world. The latest data show that there were nearly 1.3 million new cases of prostate cancer and 359,000 prostate cancer-related deaths worldwide in 2018, being the fifth largest cause of death in men^1^. PSA is secreted by prostate epithelial cells and is a widely used biomarker for the early diagnosis of prostate cancer^2^. Immunoassay methods have been most widely used in the detection of PSA, such as enzyme-linked immunosorbent assay (ELISA)^3,4^, radioimmunoassay (RIA)^5^, chemiluminescence immunoassay (CLIA)^6^, and fluorescent immunoassay (FIA)^7^. However, these methods are burdened with limitations such as low sensitivity, complicated operations, and a lack of amenability with automatic detection. Thus, it is necessary to develop a rapid, sensitive, and automatic platform for PSA detection. An electrochemical biosensor is one such assay and is a simple, inexpensive, and accurate method for PSA measurement^8–11^.

One of the critical problems that should be solved for electrochemical detection is to improve its sensitivity. Many strategies have been used to improve the sensitivity of electrochemical biosensors, such as chemical derivatization, enzymatic reaction, and nanomaterial modification^12–17^. Among these strategies, nanomaterial modification has attracted increasing interest in recent years because it can improve the sensitivity of electrochemical biosensors by regulating the electrochemical interface through physical and chemical methods that change the structure and state of nanoparticles. One of the common strategies of nanomaterial modification is to deposit inorganic nanomaterials on electrodes. Pan et al.^18^ constructed a biosensor based on graphene oxide and dual-antibody-modified poly-l-lactide nanoparticles for PSA detection, achieving a detection limit of 1 ng/mL. The detection limit of glycated hemoglobin A1c (HbA1c) achieved in our group was 5 μg/mL with the electrodeposition of gold nanoflowers (Au NFs) onto the electrode^19^. Recently, DNA nanostructures have attracted much attention due to their high precision, structural stability, and ease of preparation^20–23^. In particular, three-dimensional DNA tetrahedron structural probes (TSPs) were introduced to provide an ideal scaffold for the detection of DNA^24^, microRNA^25^, cocaine^26^, and PSA^27^. DNA TSPs could modulate the distribution and orientation of antibodies, which could decrease nonspecific adsorption on the electrode surface, control the nanospacing of immobilized antibodies, and then increase the detection sensitivity efficiently^21,28–31^. For example, Wen et al.^26^ utilized DNA TSPs to promote the efficient identification of aptamers with cocaine, reducing the detection limit to 33 nM (~10 ng/mL), which was three to four orders of magnitude higher than that of similar works; Chen et al.^27^ fabricated an ultrasensitive electrochemical immunosensor for PSA detection using DNA TSPs and acquired a lower detection limit of 1 pg/mL, while the detection based on double-stranded DNA was 50 pg/mL.

Another critical problem that should be solved for electrochemical detection is to achieve rapid and automatic detection. Microfluidic chips can integrate an entire laboratory into a single chip with compelling advantages of reduced sample volume, short reaction times, low cost, and portability. Microfluidics has become an important tool for the automatic detection of clinically relevant biomarkers. Electrochemical detectors could match microfluidic chips in size, cost, and portability, which showed great application prospects for point-of-care testing (POCT). Recently, great effort has been made to combine microfluidic devices with electrochemical biosensors^32–39^. Oliveira et al.^32^ constructed a disposable microfluidic immunoarray device (DμID) based on a screen-printed array with eight electrodes for rapid detection of CA15-3; Yang et al.^39^ combined microfluidics with electrochemistry to achieve one-step detection of target molecules with an immune-sandwich reaction based on SPEs and obtained a detection limit of 500 pg/mL for PSA less than 30 min.

In this work, we developed a novel microfluidic–electrochemical (μFEC) detection system by introducing electrochemical biosensors, DNA TSPs, and microfluidic chips simultaneously for simple, rapid, and sensitive PSA detection. The electrochemical biosensor we constructed was based on a screen-printed electrode modified with gold nanoflowers (Au NFs) with the purpose of increasing the specific surface area and improving the chemical signal response. DNA TSPs were immobilized on the gold electrode surface by Au–S bonds to provide a stable scaffold for the immune-sandwich analytical system. Biotin-anti-PSA (biotin-Ab1) and horseradish peroxidase-anti-PSA (HRP-Ab2) were attached to the DNA TSPs to form the classical “sandwich” structure. The PDMS microfluidic channels were tightly coupled on the screen-printed electrodes directly after plasma treatment. To obtain the optimal results, the μFEC system performance was investigated and optimized in this work, and the feasibility was demonstrated through the detection of PSA in real serum samples.

## Materials and methods

### Reagents and apparatus

The DNA sequences (A, B, C, D, and linker) shown in Supplementary Table S1 were synthesized by Sangon Biotech (Shanghai) Co., Ltd. Streptavidin (SA), 3,3’,5,5’-tetramethylbenzidine (TMB)/H_2_O_2_, and tris(2-carboxyethyl)phosphine (TCEP) were purchased from Sigma-Aldrich (St. Louis, MO, USA). PSA, biotinylated anti-PSA monoclonal antibody, and PSA antibody modified with horseradish peroxidase were purchased from Shanghai Linc-Bioscience Co. Ltd. Casein, bovine serum albumin (BSA), Tween 20, and other chemicals were purchased from Sinopharm Chemical Reagent Co. Ltd. All chemical reagents were prepared with ultrapure water from a Millipore Milli-Q water purification system (18.2 MΩ cm resistivity).

An HSBS16x electrochemical workstation and multiple channel screen-printed electrodes (SPEs) were purchased from HuasenXinke (Suzhou) Nanotechnology Co. Ltd. The silicon mold used in this work with special dense and meandering microfluidic channels was obtained from the Shanghai Institute of Applied Physics, Chinese Academy of Sciences. The DOWSIL^TM^ 184 polydimethylsiloxane (PDMS) prepolymer and curing agent were purchased from Dow Corning.

Healthy human blood samples were obtained from Shanghai Pudong New District Zhoupu Hospital and centrifuged at 3000 r/m for 10 min at 4 °C, and the supernatant (serum) was collected and stored at −80 °C. Prostate cancer patient serum samples were obtained from Shanghai Ninth People’s Hospital, Shanghai JiaoTong University School of Medicine. The use of serum samples was approved by the Research Ethics Committees of hospitals. Serum samples used in this work were undiluted.

### Preparation of DNA TSPs

Five single-stranded DNAs (A, B, C, D, and linker) were dissolved in TE buffer (10 mM Tris, 1 mM EDTA, pH 8.0), yielding a final concentration of 100 μM. Then, 0.5 μL of each strand was combined with 10 μL TCEP (30 mM) and 87.5 μL TM buffer (20 mM Tris, 50 mM MgCl_2_, pH 8.0), and the resulting mixture was heated to 95 °C for 10 min and then cooled to 4 °C for 30 s using a T100^TM^ PCR Thermal Cycler. The final concentration of DNA TSPs was 0.5 μM.

### Preparation of polyacrylamide gel

Six milliliters of polyacrylamide gel (10%) was prepared with 2 mL of polyacrylamide (30%), 1.2 mL of 5× TBE, 0.75 mL of MgCl_2_, and 2.05 mL of Milli-Q water, and the resulting mixture was mixed well. In total, 60 μL of ammonium persulfate (APS) and 6 μL of N,N,N’,N’-tetramethylethylenediamine (TEMED) were added and mixed gently and quickly for further use.

### Construction of an electrochemical biosensor for PSA detection

Each electrode was electrodeposited with 70 μL of a HAuCl_4_ solution to obtain a layer of gold nanoflowers according to the reported protocol^19^. The parameters were as follows: deposition time, 150 s; scan rate, 100 mV/s; and deposition potential, −200 mV. SPEs were placed in a wet box to avoid the electrode surface being dried during the reaction, and the working electrodes were incubated with 5 μL of DNA TSPs (0.5 μM) overnight at room temperature. Electrodes were rinsed with PBS (pH 7.4). Each electrode was soaked in 50 μL of blocking buffer (1% casein and bovine serum albumin in PBS) at 37 °C for 2 h to eliminate nonspecific binding sites. Five microliters of SA (200 μg/mL) were linked with biotinylated DNA TSPs (Linker) at 37 °C for 30 min. After being washed with PBST, biotin-Ab1 (100 μg/mL) was linked with SA by dropping 5 μL of biotin-Ab1 at 37 °C for 1 h to form biotin-Ab1/SA/DNA TSPs/Au NFs/SPEs, and the excess reagents were washed with PBS. The resulting biosensor was stored at 4 °C for further use.

The concentrations of PSA were diluted with PBS from the initial concentration (0.5 mg/mL) to concentrations ranging from 0 to 300 ng/mL, and an HSBS16x electrochemical workstation was used to perform measurements by cyclic voltammetry (CV) and amperometry (IT). First, 5 μL PSA was coated on the working electrodes at 37 °C for 1 h. Then, 5 μL of 10 μg/mL HRP-Ab2 was added to the electrode surface at 37 °C for 1 h, and redundant reagents were washed away with PBS and dried under N_2_. Finally, each electrode was rinsed with PBST as above and soaked with 50 μL of TMB (H_2_O_2_) solution, and reactions on SPEs were detected by an electrochemical workstation. CV experiments were conducted at a scanning rate of 0.1 V/s ranging from −0.3 V to 0.7 V. The parameters of IT were as follows: initial potential, −0.1 V; run time, 300 s; sample interval, 0.5 s; current sensitivity, 1 × e^−5^ A/V; and rest time, 3 s.

### Development of the μFEC system for PSA detection

The mixtures of DOWSIL^TM^ 184 prepolymer solution and curing agent (10:1) were poured onto a silicon wafer, cured at 80 °C for 1 h, and peeled off. A shaped polydimethylsilicone (PDMS) layer was cut into individual chips, and inlet and outlet holes were punched for use. The PDMS layer was 40 mm in length and 20 mm in width, and the channels in the PDMS layer were 400 μm in width and 115 μm in height. Each electrode (10 mm width) of the SPEs used in this work comprised a carbon working electrode (3 mm diameter), a carbon counter electrode, and an Ag reference electrode (Supplementary Fig. S1). The SPEs were pretreated with biotin-Ab1/SA/DNA TSPs/Au NFs to construct an electrochemical biosensor. The modified SPEs and PDMS layer were bonded together by a YZD08-2CO_2_ plasma cleaner for 5 s (the power of the plasma cleaner was 200 W) under the protection of Eppendorf (EP) tube caps on the SPE surface. A syringe pump was used to inject PBS washing solution and other reagents into the chip through transparent microtubing, and an electrochemical workstation was used for measurement.

For PSA detection in PBS solution or spiked serum samples, the concentrations of PSA in PBS solution or healthy human serum solution were obtained by gradient dilution with concentrations ranging from 0 to 100 ng/mL. Ten microliters of PBS solution or healthy human serum solution were injected into the chip at a flow rate of 8 μL/min. With the channel and electrode surfaces washed with PBS, 10 μL HRP-Ab2 was added to the surface of electrodes at the same flow rate. After being washed again, 40 μL of TMB (H_2_O_2_) solution was injected into the chip, and PSA was detected by an electrochemical workstation. For PSA detection in real serum samples, the detection protocol was the same as in PBS solution and spiked serum samples.

## Results and discussion

### Design strategy of the electrochemical biosensor and μFEC detection system

**Scheme 1:**
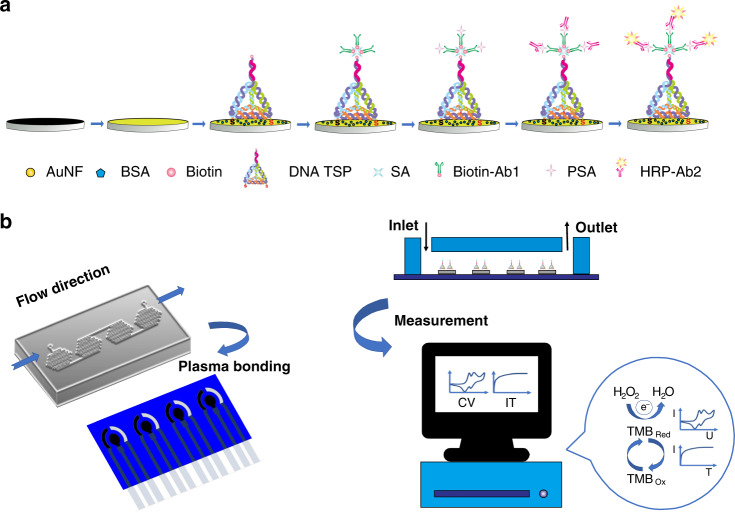
Schematic illustration of the μFEC system. **a** Scheme of the construction of the electrochemical biosensor. **b** Scheme of the development of the μFEC system for PSA detection

As shown in Scheme 1a, we first immobilized DNA TSPs onto the Au NF electrode surface through Au–S bonds^40^. Each DNA TSP consists of five single-stranded DNAs (A, B, C, D, and linker) in this work, which have a stable and rigid structure. Then, we utilized the remarkably high affinity between SA and biotin to enhance the detection sensitivity. Biotin-Ab1 was connected to the DNA TSPs through the biotin–SA interaction. The classical antibody–antigen–antibody sandwich structure was fabricated by connecting PSA and HRP-Ab2. Finally, we constructed an electrochemical biosensor with a carbon interface immune-sandwich structure mediated with DNA TSPs and obtained electrochemical signals by CV and IT, in which HRP catalyzes the reduction of H_2_O_2_ and generates signals in the presence of TMB (H_2_O_2_).

To achieve rapid and automatic PSA detection, we employed microfluidic chips to develop a μFEC detection system (Scheme 1b) and adopted dense meandering microchannels^39,41^ for sequential and stable delivery of reagents over the SPE surface. We designed a four-electrode SPE array that was bonded with the PDMS layer (Supplementary Fig. S1) by O_2_ plasma under the protection of EP tube caps on the SPE surface to avoid plasma irradiation-induced damage to antibodies. Reagents were injected into the chip with the settled volume and flow rate by the μFEC detection system. The μFEC detection system connected to the unit of liquid automatic conveying and electrochemical detection platform is shown in Supplementary Fig. S2.

### Characterization of the Au NFs-SPE and DNA TSPs

**Fig. 1:**
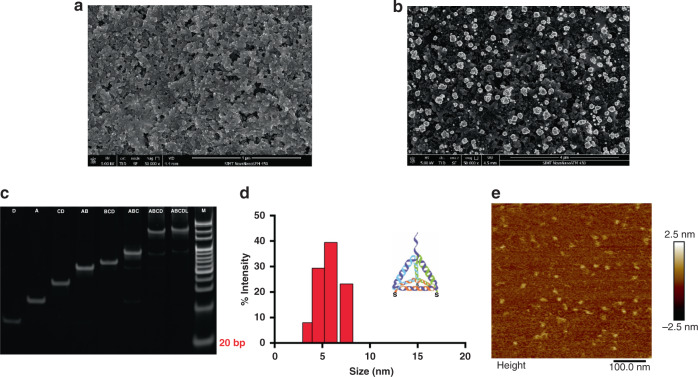
Characterization of the Au NFs-SPE and DNA TSPs. **a**, **b** SEM results of the bare carbon electrode and the electrodes after Au NF deposition. The scale value was 4 μm. **c** Gel electrophoresis image of different DNA strands. **d** DLS results of DNA TSPs. **e** AFM results of DNA TSPs

Scanning electron microscopy (SEM) was employed to characterize the surface of the SPEs. Figure 1a, b shows the SEM results of the bare carbon electrodes and gold nanoflower-modified electrodes. It was obvious that the carbon electrode had a relatively smooth surface, while the Au NF-modified electrodes had a highly rough surface area. After detailed analysis, we found that Au NFs were widely distributed on the electrode surface and that the diameter of the particles was approximately 78.831 nm, ranging from 7 to 237 nm. Electrodes modified with Au NFs could increase the specific surface area and improve the performance of electron transfer and chemical signal response.

We selected two single-stranded DNAs (A, D), two double-stranded DNAs (AB, CD), two triple-stranded DNAs (ABC, BCD), one four-stranded DNA (ABCD), and one five-stranded DNA (ABCDL) to assemble tetrahedra, and the formation of the tetrahedron in solution was confirmed through polyacrylamide gel electrophoresis (PAGE). As shown in Fig. 1c, negatively charged DNA molecules move toward the anode in the electric field, and the molecular weights of eight lanes are in descending order, which is close to the theoretical value. DNA TSPs shifted slower than the combinations of triple-strand, double-strand and single-strand DNA, which confirmed that the successful formation of the DNA TSPs as the size and configuration of the DNA molecule influenced the migration speed. Dynamic light scattering (DLS) and atomic force microscopy (AFM) were then performed to characterize the DNA TSPs. Each tetrahedron is assembled from three 55-base, one 80-base, and one 15-base oligonucleotide, which form the structure with six 17-base pair edges (each base pair was approximately 0.34 nm) and one double-strand branch^42^. As shown in Fig. 1d, the size of the nanostructure acquired from DLS was approximately 5.823 nm, which was close to the calculated value (5.78 nm). The triangle in the AFM image (Fig. 1e) shows that the tetrahedral structure was formed successfully. All these results showed the correct formation of DNA TSPs.

### Optimization of the experimental conditions of the μFEC detection system

Some experimental conditions influencing the performance of the μFEC detection system were optimized. The optimization of these conditions was indicated by the signal-to-noise ratio (SNR = Current_PSA-10ng/mL_/Current_blank_) at a concentration of 10 ng/mL PSA, which was close to the value of clinical diagnostic criteria for prostate cancer^8,43^.

**Fig. 2:**
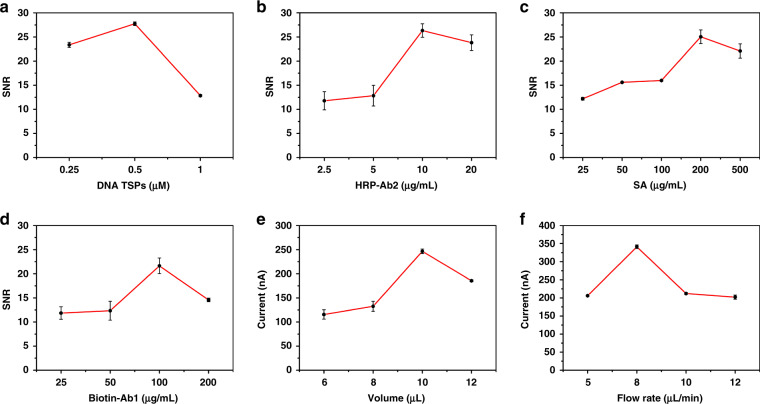
Optimization of the experimental conditions of the μFEC detection system. Optimization of the concentrations of (**a**) DNA TSPs and (**b**) HRP-Ab2 (**c**) SA (**d**) biotin-Ab1 and the conditions of (**e**) volume and (**f**) flow rate of μFEC detection system. The error bars show the standard deviation of four replicate determinations

DNA TSP played a key role in providing the scaffold of the sandwich structure, and HRP-Ab2 benefited the formation of sandwich structure and signal catalysis, which directly affected the analytical performance of immunoassays. As shown in Fig. 2a, the SNR reached a maximum at a concentration of 0.5 μM and was higher than that at 0.25 μM or 1 μM, suggesting that the optimal concentration of DNA TSPs was 0.5 μM. In Fig. 2b, the SNR increased significantly when the HRP-Ab2 concentration increased. However, when the concentration of HRP-Ab2 was higher than 10 μg/mL, the SNR decreased slightly, and 10 μg/mL was chosen as the optimal concentration of HRP-Ab2. The affinity between biotin and SA was also critical, which influenced the stability of the sandwich structure. A sufficient and appropriate concentration of biotin-Ab1 could ensure that PSA was captured effectively on the electrode surface. Here, the concentration of SA and biotin-Ab1 was optimized. Figure 2c shows that the SNR increased gradually with increasing SA concentration and reached a maximum at 200 μg/mL, which was higher than that of other concentrations. Similarly, in Fig. 2d, we found that the SNR increased to the maximum at 100 μg/mL biotin-Ab1 and was higher than that at 25, 50, or 200 μg/mL. Excess SA and biotin-Ab1 lowered the binding efficiency conversely, and we selected 200 μg/mL and 100 μg/mL as the optimal concentrations of SA and biotin-Ab1, respectively, to ensure that the reaction was completely finished.

In addition to the above four conditions, the volume and flow rate of reagents would also impact the μFEC detection system, which influenced the binding efficiency of antigen-antibody and the stability of the sandwich structure in the system. Based on the optimized concentrations of DNA TSP, HRP-Ab2, SA, and biotin-Ab1, the optimization of these two conditions was subsequently indicated by the current at a PSA concentration of 100 ng/mL. Figure 2e shows that a 10 μL sample volume produced a maximal signal for PSA detection compared to other sample volumes. Then, 10 μL was selected as the optimal volume for further use. Similarly, we found that a flow rate of 8 μL/min led to sufficiently strong signals within a reasonable time (Fig. 2f), while other flow rates (5, 10, and 12 μL/min) could cause an inadequate response with a lower detection current, suggesting that 8 μL/min was the optimal flow rate of the system.

### Analytical performance of the μFEC detection system

**Fig. 3:**
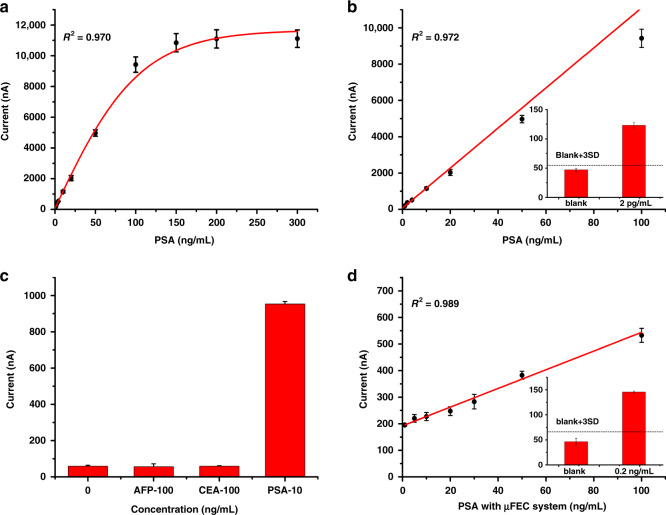
Analytical performance of the μFEC detection system. **a** The nonlinear calibration curve for PSA detection in PBS with concentrations of 0, 0.002, 0.003, 1, 2, 4, 10, 20, 50, 100, 150, 200, and 300 ng/mL by the electrochemical biosensor. **b** Linear calibration curve for PSA detection in PBS at concentrations of 0, 0.002, 0.003, 1, 2, 4, 10, 20, 50, and 100 ng/mL by the electrochemical biosensor. Inset: Histogram showing the LOD of PSA detection by the electrochemical biosensor, and the dashed lines represent the threshold (blank + 3 SD). **c** Specificity study of the electrochemical biosensor. **d** The linear calibration curve for PSA detection in PBS at concentrations of 1, 5, 10, 20, 30, 50, and 100 ng/mL with a μFEC detection system. Inset: Histogram showing the LOD of PSA detection with the μFEC detection system, and the dashed lines represent the threshold (blank + 3 SD). The error bars show the standard deviation of four replicate determinations

Under the optimized conditions determined above, we performed PSA detection in PBS by the electrochemical biosensor. First, the signal increased gradually with increasing PSA concentration and remained stable when the concentration was higher than 100 ng/mL (Fig. 3a). Then, a linear relationship between the current and the PSA concentration from 0 to 100 ng/mL was acquired (Fig. 3b). The linear regression equation was expressed as current = 110.274*[PSA] + 74.134 with a correlation coefficient of 0.972. As the signal at a concentration of 2 pg/mL was obviously higher than the threshold, which was equal to the blank control signal plus three standard deviations (3 SD), a limit of detection (LOD) of 2 pg/mL could be obtained (Fig. 3b, inset). The high detection sensitivity benefited greatly from the introduction of Au NFs, DNA TSPs, and immune-sandwich structures, which were three orders of magnitude higher than 4 ng/mL, indicating a risk of prostate cancer according to the previous works^8,43^.

The specificity test for PSA (10 ng/mL) was also carried out against alpha-fetoprotein (AFP, 100 ng/mL) and carcinoembryonic antigen (CEA, 100 ng/mL). As shown in Fig. 3c, it was obvious that the signal was as low as that of the blank compared to the higher concentrations of AFP and CEA, but it exhibited a strong signal response when a lower concentration of PSA was detected. In the presence of PSA, the current of the biosensor increased significantly, which benefited from the stable electrochemical signal generated by the classical sandwich structure of antibody–antigen-antibody. The results showed that the biosensor had a better discrimination ability for PSA than AFP and CEA.

To further realize rapid and automatic detection, we performed PSA assays using the μFEC system. In Fig. 3d, the results indicated that the regression equation was current = 3.517*[PSA] + 192.228 with a correlation coefficient of 0.989. The calibration curve of PSA in PBS solution was in the concentration range of 1–100 ng/mL with an LOD of 0.2 ng/mL (Fig. 3d, inset). The reason for the lower sensitivity of the μFEC system was that we sacrificed the longer incubation time and the larger reagent volume in exchange for a rapid test with less manual operation within 25 min.

We also compared the performance of the μFEC system with some microfluidic immunoassay methods for PSA detection (Table 1). Our μFEC system showed an obviously lower limit of detection than that of the chip-enzyme immunoassay platform^44^. The high sensitivity we achieved was attributed to the use of SPEs modified with Au NFs and DNA TSPs. Compared with other microfluidic immunoassay methods with similar sensitivity^39,45–47^, our system had a wider linear range, which allowed the detection of complicated and high concentrations of samples. In comparison to other methods in PSA detection^39,44–47^, our method integrated liquid conveying, immunoreaction, and detection in one system and accomplished all steps in less than 25 min. Based on these advantages, our μFEC system meets the requirement of clinical POCT.

**Table 1:** Comparison of the μFEC detection system with other methods for PSA detection

Method	Dynamic range (ng/mL)	LOD (ng/mL)	Reaction time (min)	Reference
An intelligent microscale electrochemical device	0.5–100	0.5	30	^39^
Microfluidic chip-enzyme immunoassay	3.2–50	3.2	30	^44^
A microfluidic immunoreaction platform	0.1–20	0.1	40	^45^
Microfluidic paper-based analytical device	1–50	0.3	45	^46^
Microfluidic paper-based fluorometric immunodevice	1–40	0.4	45	^47^
μFEC detection system in this work	1–100	0.2	25	

### Application to spiked serum and real serum sample analysis of the μFEC detection system

**Fig. 4:**
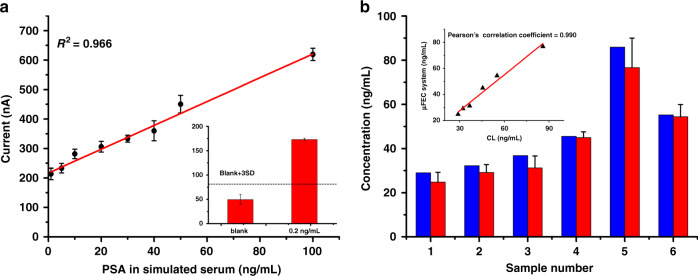
Application to complex sample analysis of the μFEC detection system. **a** The linear calibration curve of PSA detection in simulated serum with concentrations of 1, 5, 10, 20, 30, 40, 50, and 100 ng/mL by the μFEC detection system. Inset: Histogram showing the LOD of PSA detection in simulated serum, and the dashed lines represent the threshold (blank + 3 SD). **b** Comparisons between the results by CL from the hospital and our μFEC detection system. Blue columns represent the reference value by the CL, and red columns represent the detection value of the μFEC detection system. The error bars show the standard deviation of four replicate determinations. Inset: Calibration curve of correlation analysis between two methods

To investigate the clinical practical application of the μFEC detection system, we continued to detect PSA in spiked serum samples with different PSA concentrations, which were obtained by gradient dilution. As shown in Fig. 4a, the current increased gradually with increasing PSA concentration, and a linear relationship between the current and PSA concentrations from 1 ng/mL to 100 ng/mL was acquired. The linear equation was current = 4.042*[PSA] + 216.62 with a correlation coefficient of 0.966, and the limit of detection (LOD) was 0.2 ng/mL (Fig. 4a, inset). Previous reports have indicated that a PSA level of 4 ng/mL is usually applied to indicate a risk of prostate cancer^8,43^. A PSA concentration of 0.2 ng/mL is enough to monitor the recurrence of prostate cancer.

Then, we applied our μFEC detection system to detect PSA in recent real serum samples after centrifugal treatment of six prostate cancer patients with different stages. The detection results of the chemiluminescence (CL) method from the hospital and the μFEC system are shown in Supplementary Table S2. As shown in Fig. 4b, the two methods exhibited a strong correlation, with a Pearson’s correlation coefficient of 0.990 (Fig. 4b, inset). As CL is the “gold-standard” assay in hospitals for PSA detection, it was well manifested that our μFEC detection system has complicated sample analysis capability and could be used in clinical sample analysis procedures. The total reaction time was less than 25 min, which means it is useful for POCT and has potential clinical applications for routine screening of prostate cancer.

## Conclusions

In summary, we developed a novel microfluidic–electrochemical (μFEC) system for the detection of PSA. The electrochemical biosensor was first constructed by SPEs modified with Au NFs and DNA TSPs, which achieved a linear dynamic range of 0–100 ng/mL and an LOD of 2 pg/mL. Then, we developed a microfluidic–electrochemical detection system for rapid and automatic PSA detection based on microfluidic chips together with an electrochemical biosensor. Under optimized conditions, we obtained a good linear range (1–100 ng/mL) and detection limit (0.2 ng/mL) with a total reaction time of less than 25 min. Moreover, we successfully detected PSA in real clinical samples. This system we developed had the following features: (i) DNA TSPs were used as the basis to form and stabilize the sandwich structure and were in favor of decreasing the nonspecific adsorption on the SPE surface and improving the binding efficiency; (ii) classical immune-sandwich structures benefit the stable, reliable and sensitive detection of PSA; (iii) this technique was easy to use without professional skills and complicated instruments; and (iv) this method has the potential to realize the parallel detection of multiple biomarkers. On the basis of this research, we believe this system will hopefully be used for POCT and have a brilliant future for practical clinical applications.

## Supplementary information

Supplementary Information
