# Supplementary material for: DNA tetrahedron-mediated immune-sandwich assay for rapid and sensitive detection of PSA through a microfluidic electrochemical detection system
Source: Microsyst Nanoeng. 2021 Apr 25;7:33. doi: 10.1038/s41378-021-00258-x (PMC8433179; doi:10.1038/s41378-021-00258-x)
Supplement: Supplementary file 1 — Supplementary Information [file 41378_2021_258_MOESM1_ESM.docx]

**Supplementary Information**

DNA tetrahedron-mediated immune sandwich assay for rapid and sensitive detection of PSA through a microfluidic electrochemical detection system

Dezhi Feng^1,2,3#^ Jing Su^4,#^, Yi Xu^2^, Guifang He^2,5^, Chenguang Wang^2,3^, Xiao Wang^2,5^, Tingrui Pan^6^, Xianting Ding^4^ and Xianqiang Mi^1,2,3,7,8^*

^1^ Key Laboratory of Functional Materials for Informatics, Shanghai Institute of Microsystem and Information Technology, Chinese Academy of Sciences, Shanghai 200050, China.

^2^ Shanghai Advanced Research Institute, Chinese Academy of Sciences, Shanghai 201210, China.

^3^ University of Chinese Academy of Sciences, Beijing 100049, China.

^4^ School of Biomedical Engineering, Institute for Personalized Medicine, Shanghai Jiao Tong University, Shanghai 200030, China.

^5^ School of Life Sciences, Shanghai University, Shanghai, 200444, China.

^6^ Shenzhen Institutes of Advanced Technology, Chinese Academy of Science, 1068 Xueyuan Avenue, Shenzhen, 518055, China.

^7^ CAS Center for Excellence in Superconducting Electronics, (CENSE), Shanghai 200050, China.

^8^ Key Laboratory of Systems Biology, Hangzhou Institute for Advanced Study, University of Chinese Academy of Sciences, Chinese Academy of Sciences, Hangzhou 310024, China

*Corresponding author.

*E-mail address:* [mixq@mail.sim.ac.cn](mailto:mixq@mail.sim.ac.cn) (Xianqiang Mi)

^#^ Equal contributing authors.

**Table S1.** DNA sequences used in the work.

|  | Sequence (from 5’ to 3’) |
| --- | --- |
| A | ACA TTC CTA AGT CTG AAA CAT TAC AGC TTG CTA CAC GAG AAG AGC CGC CAT AGT ATT TTT TTT TTG TAT CCA GTG GCT CA |
| B | HS-TAT CAC CAG GCA GTT GAC AGT GTA GCA AGC TGT AAT AGA TGC GAG GGT CCA ATA C |
| C | HS-TCA ACT GCC TGG TGA TAA AAC GAC ACT ACG TGG GAA TCT ACT ATG GCG GCT CTT C |
| D | HS-TTC AGA CTT AGG AAT GTG CTT CCC ACG TAG TGT CGT TTG TAT TGG ACC CTC GCA T |
| Linker | biotin-TGA GCC ACT GGA TAC |

**Table S2.** Comparison of results of chemiluminescence (CL) method from hospital and μFEC detection system.

| Patients serum sample number | Chemiluminescence (CL) method from hospital (ng/mL) | μFEC detection system in this work (ng/mL) | Standard Deviation  (± SD) |
| --- | --- | --- | --- |
| 1 | 29.00 | 24.79 | 3.60 |
| 2 | 32.24 | 29.12 | 2.95 |
| 3 | 36.81 | 31.21 | 4.42 |
| 4 | 45.55 | 44.96 | 2.14 |
| 5 | 85.93 | 76.65 | 10.90 |
| 6 | 55.19 | 54.36 | 4.51 |


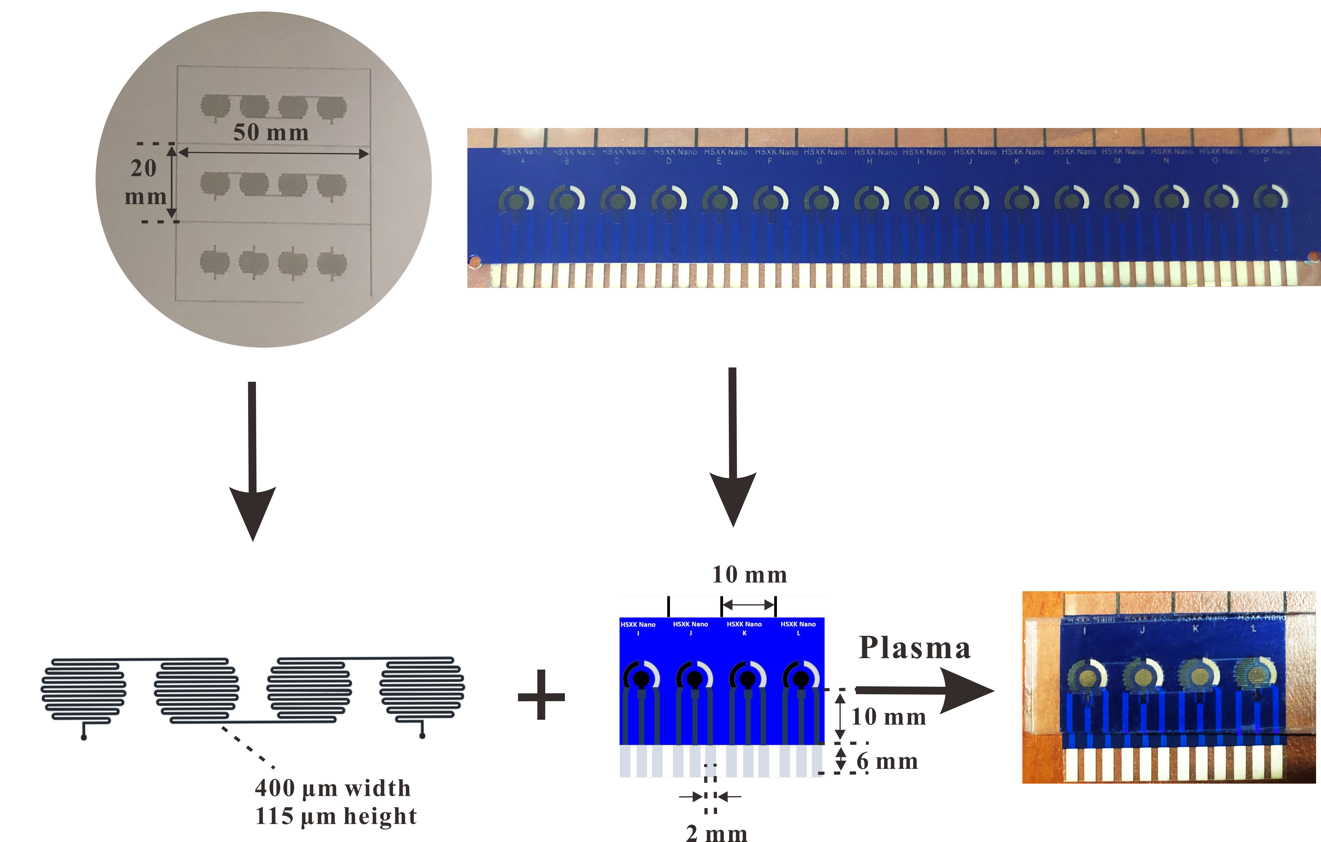


**Fig. S1.** Silicon mold with designed microchannels and multiple channel screen-printed electrodes used in this work with detailed sizes.


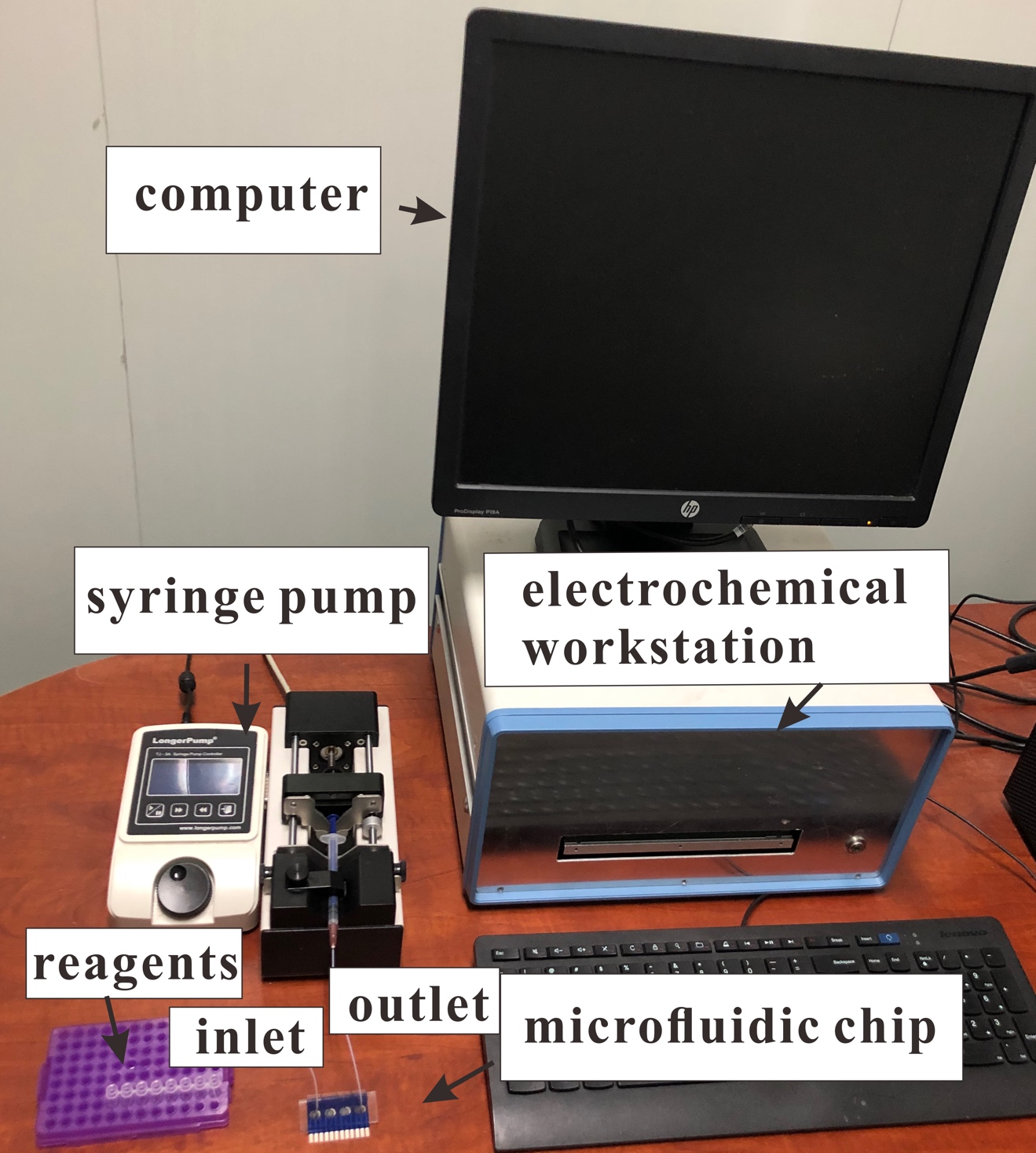


**Fig. S2.** The whole devices of μFEC system.
